# Mercury in Pancreatic Cells of People with and without Pancreatic Cancer

**DOI:** 10.3390/ijerph17238990

**Published:** 2020-12-02

**Authors:** Roger Pamphlett, Andrew J. Colebatch, Philip A. Doble, David P. Bishop

**Affiliations:** 1 Discipline of Pathology, Brain and Mind Centre, Sydney Medical School, The University of Sydney, Sydney 2050, Australia; 2 Department of Neuropathology, Royal Prince Alfred Hospital, Sydney 2050, Australia; 3 Department of Tissue Pathology and Diagnostic Oncology, Royal Prince Alfred Hospital, Sydney 2050, Australia; Andrew.Colebatch@health.nsw.gov.au; 4 Elemental Bio-Imaging Facility, School of Mathematical and Physical Sciences, University of Technology Sydney, Sydney 2007, Australia; Philip.Doble@uts.edu.au (P.A.D.); David.Bishop@uts.edu.au (D.P.B.)

**Keywords:** pancreatic cancer, mercury, carcinogenesis, elemental analysis, pancreatic ductal adenocarcinoma, toxic metal, risk factor, environmental toxicity, heavy metal, cadmium

## Abstract

Toxic metals have been implicated in the pathogenesis of pancreatic cancer. Human exposure to mercury is widespread, but it is not known how often mercury is present in the human pancreas and which cells might contain mercury. We therefore aimed to determine, in people with and without pancreatic cancer, the distribution and prevalence of mercury in pancreatic cells. Paraffin-embedded sections of normal pancreatic tissue were obtained from pancreatectomy samples of 45 people who had pancreatic adenocarcinoma, and from autopsy samples of 38 people without pancreatic cancer. Mercury was identified using two methods of elemental bio-imaging: (1) With autometallography, inorganic mercury was seen in islet cells in 14 of 30 males (47%) with pancreatic cancer compared to two of 17 males (12%) without pancreatic cancer (*p* = 0.024), and in 10 of 15 females (67%) with pancreatic cancer compared to four of 21 females (19%) without pancreatic cancer (*p* = 0.006). Autometallographic mercury was present in acinar cells in 24% and in periductal cells in 11% of people with pancreatic cancer, but not in those without pancreatic cancer. (2) Laser ablation-inductively coupled plasma-mass spectrometry confirmed the presence of mercury in islets that stained with autometallography and detected cadmium, lead, chromium, iron, nickel and aluminium in some samples. In conclusion, the genotoxic metal mercury is found in normal pancreatic cells in more people with, than without, pancreatic cancer. These findings support the hypothesis that toxic metals such as mercury contribute to the pathogenesis of pancreatic cancer.

## 1. Introduction

Worldwide, pancreatic cancer is the twelfth most common cancer, with the seventh-highest mortality rate [1]. Only 5–10% of pancreatic cancer patients have a family history of pancreatic cancer [2], so the search for environmental triggers for this cancer continues to aid interventions for prevention and early detection [1]. Environmental factors considered to increase the risk of pancreatic cancer include smoking, a high fasting plasma glucose, a high body-mass index, chronic pancreatitis, and high alcohol consumption [1,3].

**Figure 1:**
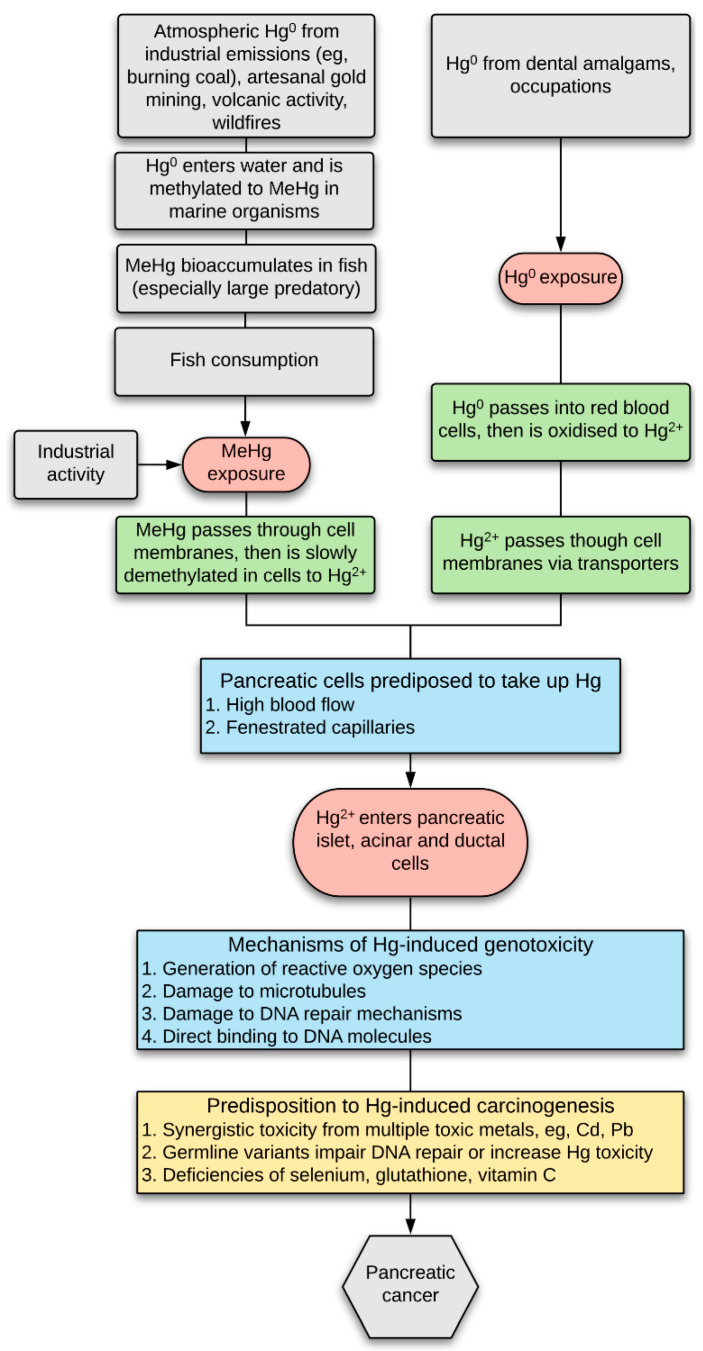
Hypothesis of mercury-induced pancreatic cancer. Mercury (Hg) exposure in humans is common due to the uptake of either (1) methylmercury (MeHg), which is slowly converted in cells to more toxic divalent mercury cations (Hg^2+^), and (2) mercury vapour (Hg^0^) which is converted to Hg^2+^ in red blood cells and then passes into cells via transporters. Pancreatic islets, and nearby acinar and ductal cells, are susceptible to the uptake of xenobiotics since they have a high blood flow and fenestrated capillaries. Mercury can trigger carcinogenesis either through the production of oxygen free radicals, damage to microtubules and DNA repair mechanisms, or direct damage to DNA [10]. People are more predisposed to mercury-induced carcinogenesis if they have been exposed to multiple toxic metals (including Cd and Pb), if they have germline variants that reduced DNA repair or increased mercury toxicity, or if they have deficiencies of selenium or other anti-mercury defences.

Genotoxic metals such as cadmium have also been proposed to play a part in the pathogenesis of a variety of cancers [4], including pancreatic cancer [5,6,7,8,9]. Mercury is another heavy metal that numerous experimental studies indicate has genotoxic potential, with recent reviews indicating that mercury causes DNA damage via multiple molecular mechanisms [10,11] (Figure 1). For example, mercury binds tightly to DNA and causes single-strand breaks in DNA which are not repaired [12], it promotes the formation of reactive oxygen species that damage DNA either directly or via forming DNA reactive products, and it damages DNA repair enzymes, DNA polymerases, and microtubules (reviewed in [10,11]). In humans exposed to mercury occupationally or via amalgam fillings, peripheral lymphocyte studies indicate a genotoxic effect of mercury, and several human studies have suggested increased cancer rates in people who have been exposed to mercury (reviewed in [10]). Humans are commonly exposed to both methylmercury and mercury vapour, which are metabolised in the body to toxic divalent mercury cations that accumulate in cells [13] (Figure 1). Particularly in people who have genetic and other susceptibilities to mercury toxicity, divalent mercury cations have the potential to trigger genetic changes that lead to cancer (Figure 1). However, the prevalence and distribution of mercury in the human pancreas is not known.

The proposal that a direct link exists between mercury exposure and human cancers remains controversial [4]. One obviously cannot intentionally expose people to mercury and see what cancers they develop years later, but indirect evidence for such a link can be sought by looking for mercury in normal pancreatic tissue adjacent to pancreatic tumours, and seeing if mercury here is more common than in people who did not have pancreatic tumours. Determining the elemental composition of human pancreatic cells is difficult, since islet cells make up only a small proportion of the total cell number, and mercury in single or a small number of pancreatic cells could initiate a pancreatic neoplasm. To overcome this problem, we employed two techniques that allow the in situ localisation of mercury within tissues, i.e., the histochemical technique of autometallography which detects intracellular mercury [14,15], and laser ablation-inductively coupled plasma-mass spectrometry (LA-ICP-MS) which detects multiple elements [16]. We therefore studied pancreas samples containing normal pancreatic tissue from people who had had surgery for pancreatic cancer and compared their pancreatic mercury distribution, and how often they had pancreatic mercury, to people who did not have pancreatic cancer.

## 2. Materials and Methods

### 2.1. Ethics

This study (X14-029) was approved by the Human Research Committee, Sydney Local Health District (Royal Prince Alfred Hospital Zone), and by the office of the New South Wales Coroner. The institutional review board waived the need for written informed consent from patients or relatives of deceased individuals studied since this was a de-identified retrospective study of archived paraffin-embedded tissue.

### 2.2. Sample Collection

Paraffin sections of the pancreas containing at least 10 normal islets were obtained from two sources: (1) The Sydney Royal Prince Alfred Hospital tissue archive supplied paraffin blocks from pancreatectomies for pancreatic ductal adenocarcinoma (not otherwise specified) taken from 30 males (mean age 69 years, age range 39–87 years, SD 13 years), and 15 females (mean age 69 years, age range 39–83 years, SD 13 years). De-identified tissue histopathology reports for these cases were obtained from the Department of Tissue Pathology and Diagnostic Oncology at Royal Prince Alfred Hospital. (2) The New South Wales Department of Forensic Medicine tissue archive supplied paraffin blocks of pancreas taken as part of routine sampling from autopsies of people who did not have pancreatic cancer, comprising 17 males (mean age 63 years, age range 35–96 years, SD 24 years), and 21 females (mean age 74 years, age range 35–104 years, SD 27 years). The autopsy cases were selected from 103 pancreas samples in total, but 65 of these had post mortem autolytic changes and so were not suitable for analysis. The Department of Forensic Medicine supplied de-identified information on the age, gender, major pre-mortem clinical conditions, and causes of death for these samples. Major medical conditions of the autopsy cases were: none known (*N* = 16), neurodegenerative disease (*N* = 16), psychosis (*N* = 4), and one each of epilepsy and anorexia nervosa. Causes of death of the autopsy cases were: cardiovascular (*N* = 11), suicide (*N* = 7), infection (*N* = 7), trauma (*N* = 5), drowning (*N* = 3), drug overdose (*N* = 2), and one each of lung cancer, undernutrition, and undetermined.

### 2.3. Mercury (Autometallography) Staining

Paraffin blocks were sectioned at 7 μm with a Feather S35 stainless steel disposable microtome blade and deparaffinised. Sections were stained for inorganic mercury using silver nitrate autometallography, which represents the presence of mercury as black silver grains surrounding the mercury [17]. Autometallography is a sensitive amplification technique that can detect as few as 10 mercury sulphide/selenide molecules in a cell [18]. Briefly, sections were placed in a physical developer containing 50% gum arabic, citrate buffer, hydroquinone and silver nitrate at 26 °C for 80 min in the dark then washed in 5% sodium thiosulphate to remove unbound silver. Sections were counterstained with mercury-free hematoxylin and viewed with bright-field microscopy. Each staining run included a control section of a mouse spinal cord where motor neuron cell bodies contained mercury following an intraperitoneal injection of mercuric chloride [19]. Control pancreatic sections were stained with hematoxylin only. Only one pancreas tissue block was available from the autopsy cases, so for valid comparisons, only one block of the normal pancreas was examined from the pancreatectomy samples. Islets containing mercury were identified using a 10 × 10 eyepiece grid viewed at 200× magnification stepped sequentially throughout one autometallography-stained section. A mercury-stained islet was defined as an islet (surrounded by normal acinar cells) where any cells contained more than 5 autometallography grains. The proportion of autometallography-stained islets was categorised as + if fewer than 50% of islets stained positively, and ++ when 50% or more islets stained positively. The number and density of islets varied between individual samples, and mercury-stained islets were usually patchy in distribution and present in small groups, so it was considered appropriately conservative to compare the absence/presence of mercury-containing islets between pancreatic cancer and non-cancer groups.

### 2.4. Laser Ablation-Inductively Coupled Plasma-Mass Spectrometry (LA-ICP-MS)

To confirm which metal autometallography was demonstrating (since autometallography can also detect inorganic silver and bismuth), 7 μm paraffin sections of selected pancreas samples were deparaffinised and subjected to LA-ICP-MS for mercury, silver, bismuth, aluminium, gold, cadmium, chromium, iron, nickel and lead, as well as for zinc to localise islets due to their high zinc levels [20]. Analyses were carried out on a Teledyne Cetac LSX-213 G2+ laser (Omaha, NE, USA) hyphenated to an Agilent Technologies 8900 ICP-MS (Santa Clara, CL, USA), with argon used as the carrier gas. LA-ICP-MS conditions were optimised on NIST 612 Trace Element in Glass CRM (US Department of Commerce, Gaithersburg, MD, USA) and the sample was ablated with a 50 µm spot size and a scan speed of 100 µm/s at a frequency of 20 Hz. The data were collated into a single image file using in-house developed software and visualised using FIJI open source image processing (LOCI, University of Wisconsin, WI, USA).

### 2.5. Statistical Analyses

Prism v8.4 software (GraphPad, San Diego, CL, USA) was used to compare categorical variables with contingency analyses and Fisher’s exact test.

## 3. Results

### 3.1. Mercury (Autometallography) Staining

#### 3.1.1. Islet Cells

**Figure 2:**
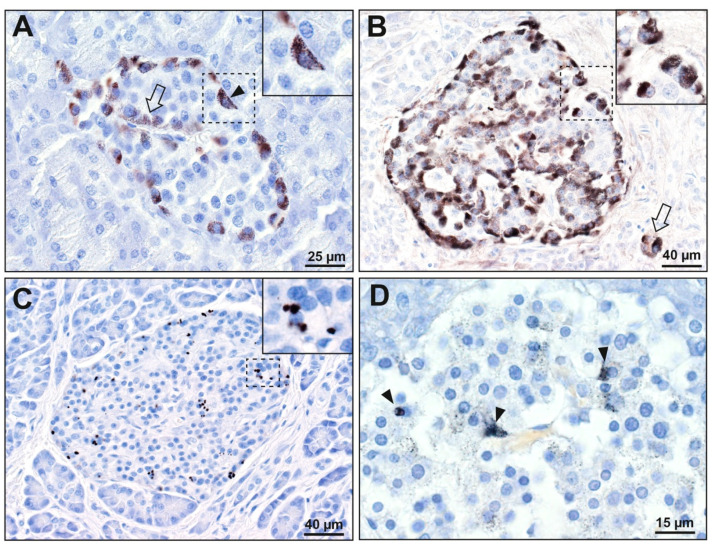
Mercury in pancreatic islet cells. (**A**–**C**): pancreatectomy samples. (**D**): autopsy sample. The areas in the dashed rectangles are shown at higher magnifications in the insets. (**A**) Black mercury grains are present in most peripheral cells (e.g., arrowhead) of this islet, as well as in a few internal cells adjacent to microvessels (e.g., arrow). (**B**) Black mercury grains in this islet are present in the cytoplasm of all peripheral cells, as well as in most internal cells adjacent to microvessels. Two cells outside the islet (arrow, probably ectopic islet cells) contain mercury. (**C**) Numerous dense black mercury granules are visible within peripheral and internal cells of this islet, usually adjacent to cell nuclei. (**D**) Fine black mercury grains are present in many scattered cells of this islet, with denser staining in three cells (arrowheads). Autometallography/hematoxylin.

Islet cells were the most prone of the pancreatic cells to contain mercury (Table S1). Mercury was present in the islets of 30 of the 83 samples (36%) from both pancreatectomy and autopsy groups combined. In people with pancreatic cancer, 24 of 45 (53%) had islets that stained for mercury. The most common distribution of mercury in these islets was in the cytoplasm of peripheral islet cells, with either a few or many internal islet cells also containing mercury (Figure 2). Islet cells that were adjacent to microvessels, either the peripheral cells next to the circumferential vessels or the internal cells adjacent to the interior vessels, were most likely to contain mercury. In all mercury-positive samples, combinations of islets with or without mercury were seen. Mercury-positive islets were often arranged in small clusters, with other islets being mercury-negative. In four of the pancreatectomy samples, 50% or more islets contained mercury (Table S1).

Six of 38 people (16%) without pancreatic cancer had mercury-stained islets (Table S1). In five of these, the mercury staining was mostly faint (Figure 2), and involved fewer than 50% of islets. Only one autopsy sample had mercury staining involving more than 50% of islets.

Mercury was seen more often in islet cells in 14 of the 30 males (47%) with pancreatic cancer, compared to two of the 17 males (12%) without pancreatic cancer (*p* = 0.024), and more often in 10 of the 15 females (67%) with pancreatic cancer, compared to four of the 21 females (19%) without pancreatic cancer (*p* = 0.006).

To look for any effect of ageing on mercury in the pancreas, the proportion of the 83 people (in both groups combined) with mercury-containing islets was calculated. The proportion of people in increasing age quartiles with islet mercury was: 35–50 years: 43%, 51–72 years: 48%, 73–83 years: 36%, and 84–104 years: 12%. Therefore, the mercury did not appear to accumulate in the pancreas on ageing. The small proportion of mercury-containing islets in the 84–104 years group is likely to be due to the larger proportion of non-pancreatic-cancer cases (88%) in this group.

Gender did not appear to affect pancreatic uptake of mercury in the 83 people of both groups combined, with 16 of the 47 males (34%) having mercury-containing islets, compared to 14 of the 36 females (39%) (*p* = 0.65).

There were not enough numbers in subgroups of non-pancreatic-cancer premortem medical conditions or causes of death to undertake reliable statistical analyses of the prevalence of mercury in these subgroups.

#### 3.1.2. Acinar Cells

**Figure 3:**
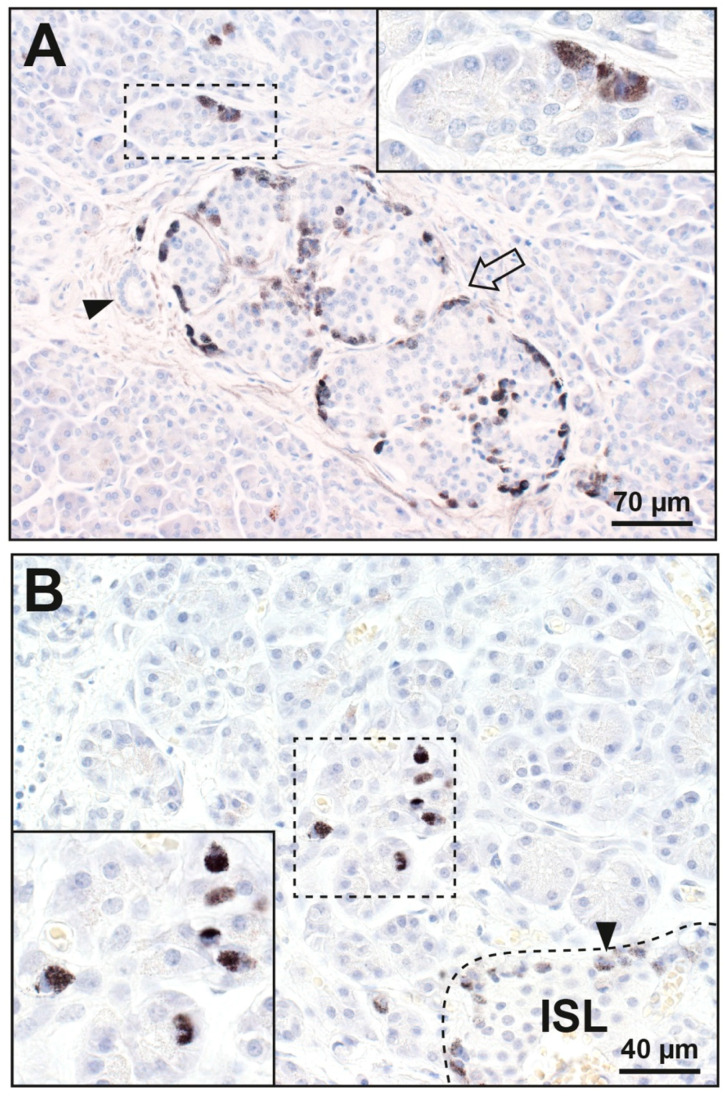
Mercury in pancreatic acinar cells. The areas in the dashed rectangles are shown at higher magnifications in the insets. (**A**) Some acinar cells (e.g., in the inset) contain black mercury grains. A nearby large islet (arrow) has mercury-positive peripheral and internal cells. A small duct (arrowhead) adjacent to the islet does not contain mercury. (**B**) A group of glands (within the dashed rectangle) have acinar cells containing cytoplasmic mercury (inset). A nearby islet (ISL, within the dashed line in the right lower corner) has peripheral cells (e.g., arrowhead) that contain mercury. Autometallography/hematoxylin.

In 11 of the 45 people (24%) with pancreatic cancer (Table S1), mercury was seen in single or small groups of acinar cells, usually in glands close to mercury-containing islets (Figure 3). Acinar cells adjacent to non-mercury islets did not contain mercury. No acinar cells contained mercury in the 38 people without pancreatic cancer.

#### 3.1.3. Periductal Cells

**Figure 4:**
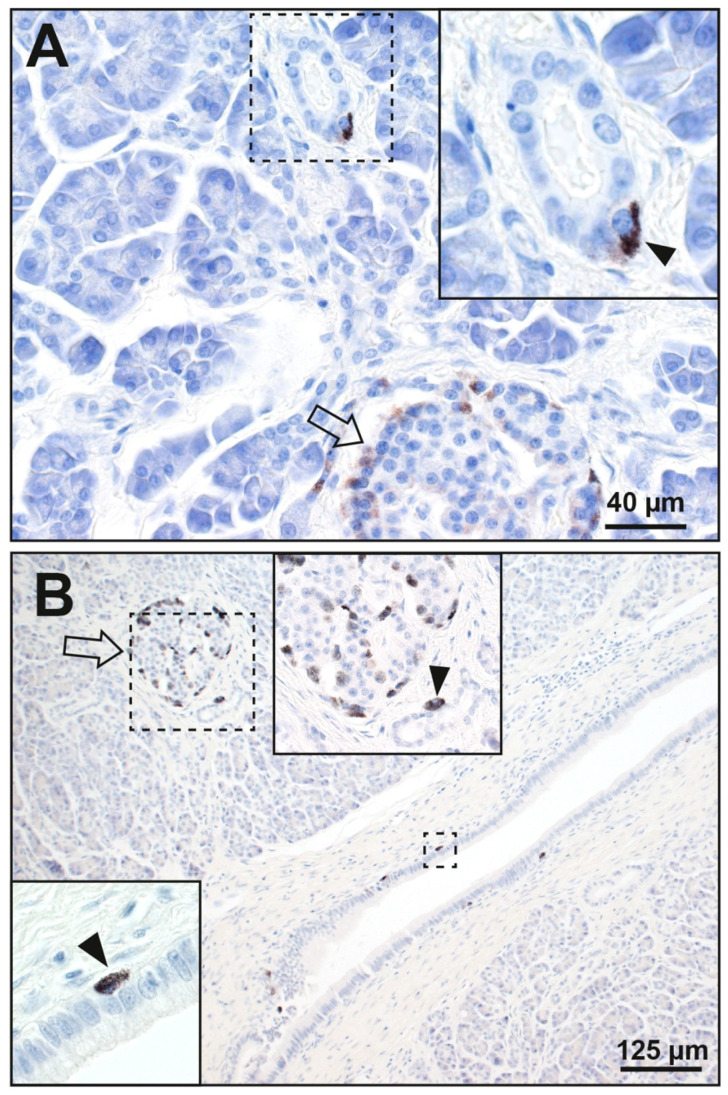
Mercury in pancreatic periductal cells. (**A**) The cytoplasm of one periductal cell abutting the epithelium of a small duct contains black mercury grains (inset, arrowhead). Some peripheral cells (arrow) of a nearby islet contain faintly-stained mercury. (**B**) Mercury is seen in scattered periductal cells adjacent to the epithelial cells of a large pancreatic duct (e.g., in the small dashed rectangle and lower left inset, arrowhead). Mercury is also seen in one cell (upper central inset, arrowhead) abutting a small duct. This is adjacent to an islet (arrow) which has peripheral and internal cells containing mercury. Autometallography/hematoxylin.

Mercury was present in periductal cells in 5 of the 45 people (11%) with pancreatic cancer (Table S1). In four of these, mercury was in cells abutting the epithelial cells of small intercalated or interlobular ducts (Figure 4), usually close to mercury-containing islets. In one of these, mercury was also seen in a few scattered oval cells abutting the epithelial cells of a larger interlobular pancreatic duct (Figure 4). No periductal cells in the 38 people without pancreatic cancer contained mercury.

### 3.2. LA-ICP-MS

#### 3.2.1. Correlation of Mercury Detected on Autometallography with LA-ICP-MS

**Figure 5:**
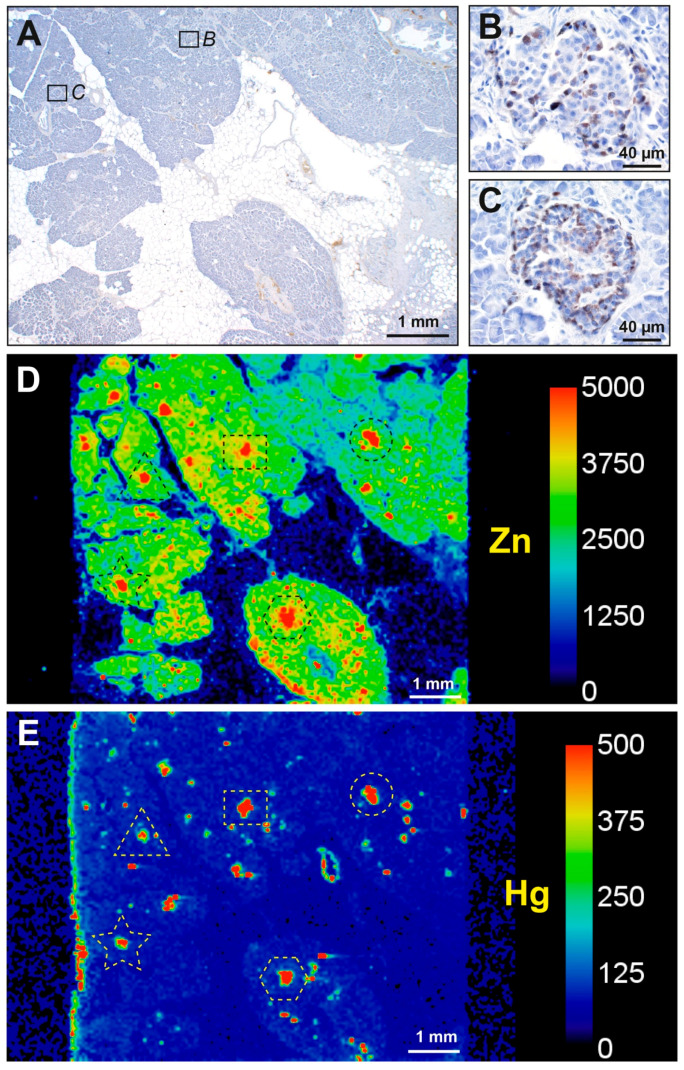
LA-ICP-MS confirmation of mercury detected by autometallography. (**A**) A low power image of an autometallography/hematoxylin-stained pancreas sample shows the exocrine tissue (blue-stained) and fatty infiltration (pale regions). (**B**,**C**) Examples of two of the many islets in the sample (**A**) whose cells stained with autometallography (i.e., black grains). Autometallography/hematoxylin. (**D**) An LA-ICP-MS zinc image demonstrates the islets as focal red regions (examples in individually-dashed profiles). Yellow-green regions show the normal cellular density of the exocrine pancreas. Dark blue spaces between cellular regions are due to fatty infiltration. (**E**) An LA-ICP-MS mercury image shows mercury (red regions) in the same islets demonstrated by the zinc imaging. Scale = counts per second (proportional to abundance).

**Figure 6:**
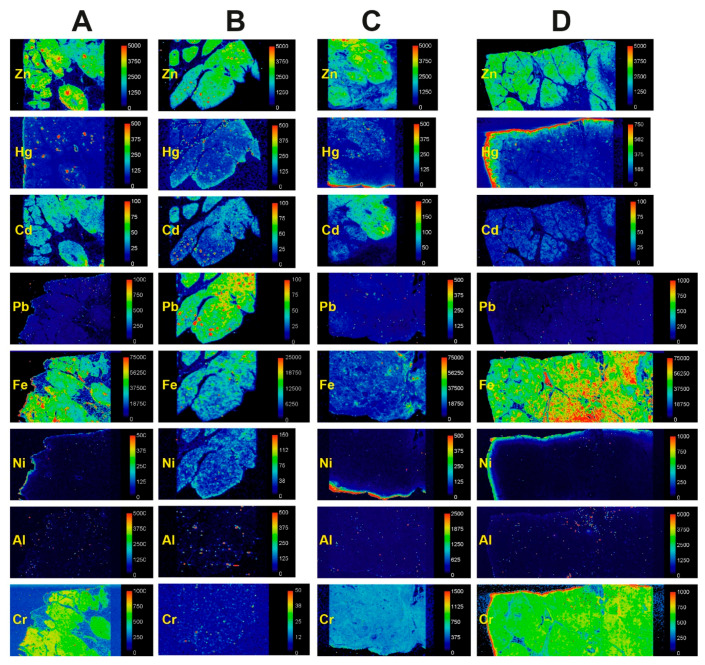
LA-ICP-MS of pancreatic samples containing AMG-positive islets. Zinc images show the overall cellularity of the tissue (yellow-green) as well as pancreatic islets (red) that contain focally higher zinc levels (some images may need to be enlarged to show these islets). (**A**) In this sample, mercury co-localises with zinc-positive islets (the top two images are shown enlarged in Figure 5). Diffuse chromium, and irregular cadmium and iron, are present. (**B**) Islet-located mercury and cadmium, focal and diffuse lead, and irregular iron, nickel, and aluminium, are present in this sample. (**C**) In this sample, islet-located mercury, diffuse chromium, and irregular cadmium and iron are present. (**D**) Islet-located mercury, diffuse chromium, and irregular iron are present in this sample. Artefactual edge effects are seen in some mercury, nickel, and chromium images (e.g., in (**D**)). Scale = counts per second (proportional to abundance).

LA-ICP-MS images showed the high zinc levels normally present in islets, as well as the overall cellularity of the sample (Figure 5). In four samples in which numerous islets stained with autometallography, zinc-detected islets co-localised with LA-ICP-MS-detected mercury (Figure 5 and Figure 6). In two autometallography-negative samples, no LA-ICP-MS mercury was seen in zinc-detected islets (Figure S1).

#### 3.2.2. Mercury and Other Toxic Metals Detected with LA-ICP-MS

In the four pancreas samples known to contain autometallography-positive islets, LA-ICP-MS images of toxic metals were classified as being either negative (including non-specific background levels and artefactual edge effects), focal (assumed to be in islets when aligned with zinc), diffuse, or irregular (Figure 6). Cadmium was seen in islets in one sample and irregularly in two. Chromium was present diffusely in three samples. Lead was present diffusely and focally in one sample. Iron was present irregularly and/or diffusely in all samples, and at higher levels within the lumen of blood vessels (presumably due to high iron levels in red blood cells). Nickel was seen irregularly, in the same distribution as iron, in one sample. Aluminium was present irregularly in one sample. In two samples with no autometallography staining (Figure S1), numerous LA-ICP-MS zinc-positive islets were seen, but no mercury was detected; cadmium was present diffusely in the pancreatic tissue of both samples, but not increased in islets. No silver, bismuth, or gold was detected in any samples.

## 4. Discussion

Key findings in this study are that mercury is present in pancreatic islet cells in a greater proportion of people with pancreatic cancer than those without pancreatic cancer, and that in several people with pancreatic cancer, individual acinar and periductal cells also contained mercury (Table S1). Other toxic metals, in particular cadmium and chromium, were found in selected samples (see Results). These findings suggest an association exists between mercury in pancreatic cells and pancreatic cancer, but as always, association does not prove causation [21]. We hope, though, our findings will promote further investigations into the role toxic metals play in the pathogenesis of pancreatic cancer.

Mercury in our pancreatic samples was found predominantly in islet cells, possibly because the plentiful capillaries that encircle and penetrate islets (which receive blood before the other pancreatic cells) are fenestrated [22,23] and so would more readily allow xenobiotics to pass into them. Some acinar and periductal cells adjacent to mercury-containing islets also contained mercury, probably because they share the same fenestrated capillaries that supply the nearby islets [24]. The preference of mercury to enter islets cells is of interest since these cells can differentiate into both pancreatic and extra-pancreatic cells, and they contain numerous carcinogen-metabolising enzymes, suggesting they play a role in carcinogenesis [25]. Mercury is a genotoxin [10,26] that has previously been implicated in the pathogenesis of neoplasms such as breast cancer [16,27]. The carcinogenic potential of mercury would be enhanced if it were present in progenitor cells since these cells when dividing are likely to be susceptible to the genotoxic properties of mercury [10,11]. However, uncertainty remains as to the location of progenitor cells in the human pancreas, with the possibility that they exist in islets, acini, or ducts, or all three locations [25,28,29,30,31,32]. Future studies combining elemental biomapping with immunostaining for progenitor and stem cell markers would be required to accurately determine the nature of these mercury-containing pancreatic cells.

The amount of mercury in the atmosphere is steadily increasing, mostly due to the burning of fossil fuels such as coal [33]. This is likely to be a factor in the increased bioaccumulation of mercury in fish [34], and seafood consumption is the major source of mercury exposure in humans [35]. Elevated blood levels of mercury have been recorded in people consuming recommended levels of seafood [36], and in avid consumers of seafood this mercury may increase markers of oxidative stress [37]. Mercury-triggered carcinogenesis might therefore be one factor contributing to the increasing incidence of pancreatic cancer that has been reported by several epidemiological studies [38], though some of this increase in incidence could be due to ageing populations [1].

There was no age-related increase in islet mercury in our combined samples. This is unlike findings in the brain [39] and pituitary [40] where greater proportions of older people had mercury-containing cells. This raises the possibility that people with pancreatic cancer are on average likely to have had a greater environmental exposure to mercury from sources such as seafood, dental amalgams, or occupations, compared to people without pancreatic cancer. Another possible explanation is that people with pancreatic cancer have a genetic susceptibility to either the cellular uptake of mercury (eg, by mercury transporters [41]) or the retention of mercury in their pancreatic cells. Pancreatic cancer could in some way change the internal milieu of the remainder of the normal pancreatic tissue, possibly by increasing blood flow, that aids the entry of mercury into pancreatic cells. Alternatively, suppressed metabolic activity in the remaining normal pancreas of people with pancreatic cancer could theoretically depress mercury excretion from the cells, thereby raising intracellular mercury levels. Future case-control studies of dietary habits, dental mercury-containing amalgam fillings [42], and occupations in people with and without pancreatic cancer are needed to shed light on whether any of these environmental sources of mercury are risk factors for pancreatic cancer. Genetic studies looking for differences in mercury toxicokinetics in people with pancreatic cancer would also be of interest [43].

Other potentially toxic metals found in our pancreatic samples were cadmium, chromium, lead, iron, nickel, and aluminium. (1) Cadmium is a genotoxin [44,45] that has been proposed to play a role in pancreatic cancer [5,6,7]. A major source of human exposure to cadmium is cigarette smoke [46], an established risk factor for pancreatic cancer [3,47], as well as rice in some countries such as Japan [48]. There is no histochemical technique to localise cadmium within individual cells, but cadmium appeared regularly in our pancreatic tissue samples studied with LA-ICP-MS. (2) High concentrations of chromium are found in the pancreatic juice of people with pancreatic carcinoma [49]. (3) An excess of pancreatic cancer mortality is found in people occupationally exposed to lead [50], and high toenail levels of lead are associated with an increased risk of pancreatic cancer [6]. (4) Iron excess is implicated in carcinogenesis due to its ability to damage DNA via the generation of oxygen radicals [51]. (5) Nickel is classified as a carcinogen [45], though high toenail levels of nickel are suggested to be associated with a lower risk of pancreatic cancer [6]. (6) There is conflicting evidence about the carcinogenicity of aluminium, though it can cross-link proteins and induce oxidative stress [52].

Mixtures of heavy metals were commonly seen in our pancreatic samples. This could be relevant to carcinogenesis since many heavy metals are classified as carcinogens [45], and toxicity-enhancing synergy between heavy metals is being increasingly recognized [53]. Cigarette smoke, in addition to containing cadmium, also contains chromium, lead and nickel [54], which accumulate in tissues after prolonged smoking [46] and could be a source of these metals found in our pancreas samples, though we did not have access to histories of smoking habits.

**Figure 7:**
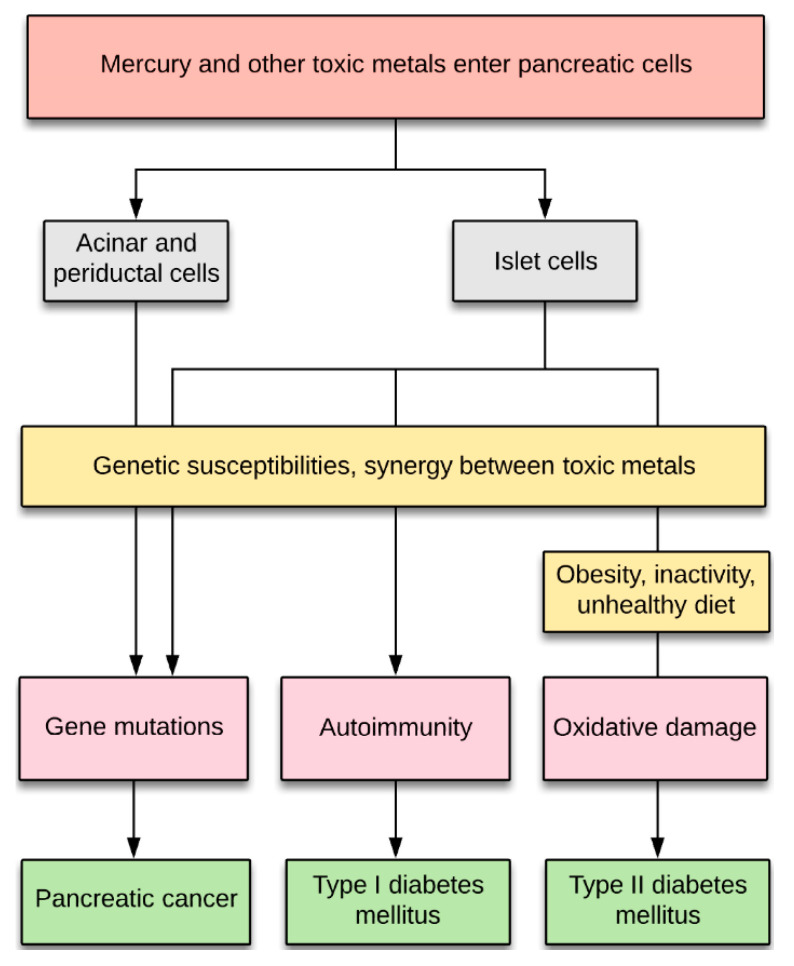
Potential contributions of toxic metals to pancreatic disorders. Exposures to toxic metals allow their entry into islet, acinar, and periductal cells. (1) Toxic metals could induce mutations in cells, aided by genetic susceptibility to metal toxicity and by synergistic effects between metals. (2) Toxic metals promoting autoimmunity in islet cells could precipitate type I diabetes mellitus in people with a genetic predisposition to immune overactivity. (3) Toxic metals causing oxidative stress could contribute to islet cell loss in type II diabetes mellitus.

Our finding that mercury is found in a variety of human pancreatic cells, often together with other toxic heavy metals, supports previous proposals that heavy metals with genotoxic [10,11,44], autoimmune [55], or oxidative effects [56] could play a part in the pathogenesis of both pancreatic cancer [5,6,7,8,9] and diabetes mellitus [57,58,59,60,61,62,63,64]. In pancreatic cancer, most attention until now has been given to the possible role of cadmium [5,7,8,9], whereas both mercury [58,59,60,63] and cadmium [64] are suspected to contribute to the pathogenesis of diabetes. Metal toxicity in these pancreatic disorders could be enhanced by synergistic actions between toxic metals [53], as well as by genetic susceptibilities to metal toxicity [65], autoimmunity [66], and oxidative damage [56] (Figure 7).

This study has limitations. (1) We did not have DNA samples to look for genetic susceptibilities to mercury toxicity [65]. Future prospective studies combining epidemiological, genetic, and pathological investigations would be required to study this association. (2) Autometallography demonstrates only the inorganic form of mercury, but since this is the proximate toxic form of mercury [13] it is likely to be the most important to detect. (3) We could not establish the post mortem delay for the autopsy cases since these were forensic (coronial) cases where the estimation of time of death is often imprecise; consequently, we selected autopsy pancreas samples that had no or minimal evidence of autolysis. (4) Theoretically, post mortem elevations in tissue sulphide levels could increase the sensitivity of autometallography and cause over-estimation of cellular mercury. However, in humans, the length of post mortem delay does not appear to affect autometallography [67], and no increase in autometallography staining was seen in our autopsy samples (compared to surgical) samples, rather the opposite was the case. (5) The range of ages in our non-pancreatic cancer group was larger than in the pancreatic cancer group. However, the finding of no age-related increase in mercury uptake into the pancreas implies this is unlikely to be a confounder. (6) We had no access to clinical data of people with pancreatic cancer, and only access to major pre-mortem findings and causes of deaths from people who had autopsies, so we are not able to undertake correlations of the mercury content of the pancreas with cancer outcomes, mercury blood levels, medication use, or other diseases. (7) We were not able to undertake functional studies looking for molecular mechanisms in pancreatic cells containing mercury that could lead to neoplastic changes, or basic studies of mercury uptake, metabolism, and excretion. Future studies are warranted given our finding of mercury within human pancreatic cells. (8) Pancreatic precursor lesions such as acinar-to-ductal metaplasia were not detected on our sections, but future bio-elemental studies concentrating on cancer precursor lesions would be of interest to see if these early lesions contained toxic metals. (9) We did not have access to the stage or location of the pancreatic tumours, so we are unable to correlate these with mercury in the pancreas.

## 5. Conclusions

In conclusion, mercury is found in normal pancreatic islet, acinar, and periductal cells more often in people with pancreatic cancer than people without this cancer. Mercury has genotoxic effects, so these findings support the hypothesis that toxic metals such as mercury contribute to the pathogenesis of pancreatic cancer. In addition, the finding of mercury and cadmium in human pancreatic islets supports proposals that these metals play a role in diabetes mellitus. Further investigations of multiple toxic metals in pancreatic cells of people with pancreatic cancer and diabetes mellitus are needed to provide more evidence for the role of toxic metals in the pathogenesis of these disorders.

## Supplementary Materials

The following are available online at https://www.mdpi.com/1660-4601/17/23/8990/s1, Figure S1: LA-ICP-MS of samples containing AMG-negative islets. Two pancreatectomy samples (A,B) were imaged with LA-ICP-MS. No zinc-positive islets contain mercury (the location of one islet is shown within the dashed circle). Cadmium in seen diffusely throughout the pancreatic tissue. Scale = counts per second (proportional to abundance). Table S1: Ages, gender, and mercury (autometallography) staining in pancreatic samples from people with and without pancreatic cancer.

## References

[1] GBD 2017 Pancreatic Cancer Collaborators (2019). The global, regional, and national burden of pancreatic cancer and its attributable risk factors in 195 countries and territories, 1990–2017: A systematic analysis for the Global Burden of Disease Study 2017. Lancet Gastroenterol. Hepatol..

[2] Klein A.P. (2012). Genetic susceptibility to pancreatic cancer. Mol. Carcinog..

[3] Tsai H.J., Chang J.S. (2019). Environmental Risk Factors of Pancreatic Cancer. J. Clin. Med..

[4] Tchounwou P.B., Yedjou C.G., Patlolla A.K., Sutton D.J. (2012). Heavy metal toxicity and the environment. Exp. Suppl..

[5] Schwartz G.G., Reis I.M. (2000). Is cadmium a cause of human pancreatic cancer?. Cancer Epidemiol. Biomark. Prev..

[6] Amaral A.F., Porta M., Silverman D.T., Milne R.L., Kogevinas M., Rothman N., Cantor K.P., Jackson B.P., Pumarega J.A., Lopez T. (2012). Pancreatic cancer risk and levels of trace elements. Gut.

[7] Chen C., Xun P., Nishijo M., Sekikawa A., He K. (2015). Cadmium exposure and risk of pancreatic cancer: A meta-analysis of prospective cohort studies and case-control studies among individuals without occupational exposure history. Environ. Sci. Pollut. Res. Int..

[8] Djordjevic V.R., Wallace D.R., Schweitzer A., Boricic N., Knezevic D., Matic S., Grubor N., Kerkez M., Radenkovic D., Bulat Z. (2019). Environmental cadmium exposure and pancreatic cancer: Evidence from case control, animal and in vitro studies. Environ. Int..

[9] Wallace D.R., Spandidos D.A., Tsatsakis A., Schweitzer A., Djordjevic V., Djordjevic A.B. (2019). Potential interaction of cadmium chloride with pancreatic mitochondria: Implications for pancreatic cancer. Int. J. Mol. Med..

[10] Crespo-Lopez M.E., Macedo G.L., Pereira S.I., Arrifano G.P., Picanco-Diniz D.L., do Nascimento J.L., Herculano A.M. (2009). Mercury and human genotoxicity: Critical considerations and possible molecular mechanisms. Pharmacol. Res..

[11] Nersesyan A., Kundi M., Waldherr M., Setayesh T., Misik M., Wultsch G., Filipic M., Mazzaron Barcelos G.R., Knasmueller S. (2016). Results of micronucleus assays with individuals who are occupationally and environmentally exposed to mercury, lead and cadmium. Mutat. Res..

[12] Costa M., Christie N.T., Cantoni O., Zelikoff J.T., Xin W.W., Rossman T.G., Suzuki T., Imura N., Clarkson T.W. (1991). DNA damage by mercury compounds: An overview. Advances in Mercury Toxicology.

[13] Clarkson T.W. (1997). The toxicology of mercury. Crit. Rev. Clin. Lab. Sci..

[14] Danscher G., Stoltenberg M., Juhl S. (1994). How to detect gold, silver and mercury in human brain and other tissues by autometallographic silver amplification. Neuropathol. Appl. Neurobiol..

[15] Danscher G., Stoltenberg M., Kemp K., Pamphlett R. (2000). Bismuth autometallography: Protocol, specificity, and differentiation. J. Histochem. Cytochem..

[16] Pamphlett R., Satgunaseelan L., Kum Jew S., Doble P.A., Bishop D.P. (2020). Elemental bioimaging shows mercury and other toxic metals in normal breast tissue and in breast cancers. PLoS ONE.

[17] Danscher G., Moller-Madsen B. (1985). Silver amplification of mercury sulfide and selenide: A histochemical method for light and electron microscopic localization of mercury in tissue. J. Histochem. Cytochem..

[18] Danscher G., Rungby J. (1986). Differentiation of histochemically visualized mercury and silver. Histochem. J..

[19] Pamphlett R., Png F.Y. (1998). Shrinkage of motor axons following systemic exposure to inorganic mercury. J. Neuropathol. Exp. Neurol..

[20] Wijesekara N., Chimienti F., Wheeler M.B. (2009). Zinc, a regulator of islet function and glucose homeostasis. Diabetes Obes. Metab..

[21] Herbert R.D. (2020). Research Note: Causal inference. J. Physiother..

[22] Liu Y.M., Guth P.H., Kaneko K., Livingston E.H., Brunicardi F.C. (1993). Dynamic in vivo observation of rat islet microcirculation. Pancreas.

[23] Jansson L., Barbu A., Bodin B., Drott C.J., Espes D., Gao X., Grapensparr L., Kallskog O., Lau J., Liljeback H. (2016). Pancreatic islet blood flow and its measurement. Ups. J. Med. Sci..

[24] Barreto S.G., Carati C.J., Toouli J., Saccone G.T. (2010). The islet-acinar axis of the pancreas: More than just insulin. Am. J. Physiol. Gastrointest. Liver Physiol..

[25] Pour P.M., Pandey K.K., Batra S.K. (2003). What is the origin of pancreatic adenocarcinoma?. Mol. Cancer.

[26] Zefferino R., Piccoli C., Ricciardi N., Scrima R., Capitanio N. (2017). Possible Mechanisms of Mercury Toxicity and Cancer Promotion: Involvement of Gap Junction Intercellular Communications and Inflammatory Cytokines. Oxid. Med. Cell Longev..

[27] Kresovich J.K., Erdal S., Chen H.Y., Gann P.H., Argos M., Rauscher G.H. (2019). Metallic air pollutants and breast cancer heterogeneity. Environ. Res..

[28] Ku H.T. (2008). Minireview: Pancreatic progenitor cells—Recent studies. Endocrinology.

[29] Noguchi H. (2010). Pancreatic stem/progenitor cells for the treatment of diabetes. Rev. Diabet. Stud..

[30] Carpino G., Renzi A., Cardinale V., Franchitto A., Onori P., Overi D., Rossi M., Berloco P.B., Alvaro D., Reid L.M. (2016). Progenitor cell niches in the human pancreatic duct system and associated pancreatic duct glands: An anatomical and immunophenotyping study. J. Anat..

[31] Huising M.O., Lee S., van der Meulen T. (2018). Evidence for a Neogenic Niche at the Periphery of Pancreatic Islets. Bioessays.

[32] Xu Y., Liu J., Nipper M., Wang P. (2019). Ductal vs. acinar? Recent insights into identifying cell lineage of pancreatic ductal adenocarcinoma. Ann. Pancreat. Cancer.

[33] Streets D.G., Devane M.K., Lu Z., Bond T.C., Sunderland E.M., Jacob D.J. (2011). All-time releases of mercury to the atmosphere from human activities. Environ. Sci. Technol..

[34] Schartup A.T., Thackray C.P., Qureshi A., Dassuncao C., Gillespie K., Hanke A., Sunderland E.M. (2019). Climate change and overfishing increase neurotoxicant in marine predators. Nature.

[35] Clarkson T.W., Magos L., Myers G.J. (2003). The toxicology of mercury—Current exposures and clinical manifestations. N. Engl. J. Med..

[36] Karimi R., Silbernagel S., Fisher N.S., Meliker J.R. (2014). Elevated blood Hg at recommended seafood consumption rates in adult seafood consumers. Int. J. Hyg. Environ. Health.

[37] Karimi R., Vacchi-Suzzi C., Meliker J.R. (2016). Mercury exposure and a shift toward oxidative stress in avid seafood consumers. Environ. Res..

[38] Rawla P., Sunkara T., Gaduputi V. (2019). Epidemiology of Pancreatic Cancer: Global Trends, Etiology and Risk Factors. World J. Oncol..

[39] Pamphlett R., Bishop D.P., Kum Jew S., Doble P.A. (2018). Age-related accumulation of toxic metals in the human locus ceruleus. PLoS ONE.

[40] Pamphlett R., Kum Jew S., Doble P.A., Bishop D.P. (2019). Elemental Analysis of Aging Human Pituitary Glands Implicates Mercury as a Contributor to the Somatopause. Front. Endocrinol. (Lausanne).

[41] Bridges C.C., Zalups R.K. (2017). Mechanisms involved in the transport of mercuric ions in target tissues. Arch. Toxicol..

[42] Parkin Kullmann J.A., Pamphlett R. (2018). A Comparison of Mercury Exposure from Seafood Consumption and Dental Amalgam Fillings in People with and without Amyotrophic Lateral Sclerosis (ALS): An International Online Case-Control Study. Int. J. Environ. Res. Public Health.

[43] Ekstrand J., Nielsen J.B., Havarinasab S., Zalups R.K., Soderkvist P., Hultman P. (2010). Mercury toxicokinetics—Dependency on strain and gender. Toxicol. Appl. Pharmacol..

[44] Joseph P. (2009). Mechanisms of cadmium carcinogenesis. Toxicol. Appl. Pharmacol..

[45] Kim H.S., Kim Y.J., Seo Y.R. (2015). An Overview of Carcinogenic Heavy Metal: Molecular Toxicity Mechanism and Prevention. J. Cancer Prev..

[46] Caruso R.V., O’Connor R.J., Stephens W.E., Cummings K.M., Fong G.T. (2013). Toxic metal concentrations in cigarettes obtained from U.S. smokers in 2009: Results from the International Tobacco Control (ITC) United States survey cohort. Int. J. Environ. Res. Public Health.

[47] Bosetti C., Lucenteforte E., Silverman D.T., Petersen G., Bracci P.M., Ji B.T., Negri E., Li D., Risch H.A., Olson S.H. (2012). Cigarette smoking and pancreatic cancer: An analysis from the International Pancreatic Cancer Case-Control Consortium (Panc4). Ann. Oncol..

[48] Tsukahara T., Ezaki T., Moriguchi J., Furuki K., Shimbo S., Matsuda-Inoguchi N., Ikeda M. (2003). Rice as the most influential source of cadmium intake among general Japanese population. Sci. Total Environ..

[49] Carrigan P.E., Hentz J.G., Gordon G., Morgan J.L., Raimondo M., Anbar A.D., Miller L.J. (2007). Distinctive heavy metal composition of pancreatic juice in patients with pancreatic carcinoma. Cancer Epidemiol. Biomark. Prev..

[50] Ilychova S.A., Zaridze D.G. (2012). Cancer mortality among female and male workers occupationally exposed to inorganic lead in the printing industry. Occup. Environ. Med..

[51] Toyokuni S. (2009). Role of iron in carcinogenesis: Cancer as a ferrotoxic disease. Cancer Sci..

[52] Klotz K., Weistenhofer W., Neff F., Hartwig A., van Thriel C., Drexler H. (2017). The Health Effects of Aluminum Exposure. Dtsch. Arztebl. Int..

[53] Wu X., Cobbina S.J., Mao G., Xu H., Zhang Z., Yang L. (2016). A review of toxicity and mechanisms of individual and mixtures of heavy metals in the environment. Environ. Sci. Pollut. Res. Int..

[54] Pappas R.S., Fresquez M.R., Martone N., Watson C.H. (2014). Toxic metal concentrations in mainstream smoke from cigarettes available in the USA. J. Anal. Toxicol..

[55] Pollard K.M., Cauvi D.M., Toomey C.B., Hultman P., Kono D.H. (2019). Mercury-induced inflammation and autoimmunity. Biochim. Biophys. Acta Gen. Subj..

[56] Klaunig J.E., Wang Z., Pu X., Zhou S. (2011). Oxidative stress and oxidative damage in chemical carcinogenesis. Toxicol. Appl. Pharmacol..

[57] Chen Y.W., Yang C.Y., Huang C.F., Hung D.Z., Leung Y.M., Liu S.H. (2009). Heavy metals, islet function and diabetes development. Islets.

[58] Roy C., Tremblay P.Y., Ayotte P. (2017). Is mercury exposure causing diabetes, metabolic syndrome and insulin resistance? A systematic review of the literature. Environ. Res..

[59] Chen K.L., Liu S.H., Su C.C., Yen C.C., Yang C.Y., Lee K.I., Tang F.C., Chen Y.W., Lu T.H., Su Y.C. (2012). Mercuric compounds induce pancreatic islets dysfunction and apoptosis in vivo. Int. J. Mol. Sci..

[60] Mozaffarian D., Shi P., Morris J.S., Grandjean P., Siscovick D.S., Spiegelman D., Hu F.B. (2013). Methylmercury exposure and incident diabetes in U.S. men and women in two prospective cohorts. Diabetes Care.

[61] Rana S.V. (2014). Perspectives in endocrine toxicity of heavy metals—A review. Biol. Trace Elem. Res..

[62] Gonzalez-Villalva A., Colin-Barenque L., Bizarro-Nevares P., Rojas-Lemus M., Rodriguez-Lara V., Garcia-Pelaez I., Ustarroz-Cano M., Lopez-Valdez N., Albarran-Alonso J.C., Fortoul T.I. (2016). Pollution by metals: Is there a relationship in glycemic control?. Environ. Toxicol. Pharmacol..

[63] Schumacher L., Abbott L.C. (2017). Effects of methyl mercury exposure on pancreatic beta cell development and function. J. Appl. Toxicol..

[64] El Muayed M., Raja M.R., Zhang X., MacRenaris K.W., Bhatt S., Chen X., Urbanek M., O’Halloran T.V., Lowe W.L. (2012). Accumulation of cadmium in insulin-producing beta cells. Islets.

[65] Andreoli V., Sprovieri F. (2017). Genetic Aspects of Susceptibility to Mercury Toxicity: An Overview. Int. J. Environ. Res. Public Health.

[66] Gregersen P.K., Olsson L.M. (2009). Recent advances in the genetics of autoimmune disease. Annu. Rev. Immunol..

[67] Pamphlett R., Kum Jew S. (2015). Different Populations of Human Locus Ceruleus Neurons Contain Heavy Metals or Hyperphosphorylated Tau: Implications for Amyloid-beta and Tau Pathology in Alzheimer’s Disease. J. Alzheimers Dis..

