# Supplementary material for: Mercury in Pancreatic Cells of People with and without Pancreatic Cancer
Source: Int J Environ Res Public Health. 2020 Dec 2;17(23):8990. doi: 10.3390/ijerph17238990 (PMC7731371; doi:10.3390/ijerph17238990)
Supplement: Supplementary file 1 [file ijerph-17-08990-s001.zip › Table S1 201007.docx]

**Table S1.** Ages, gender, and mercury (autometallography) staining in pancreatic samples from people with and without pancreatic cancer.

| **People with pancreatic cancer (pancreatectomy)** | | | | **People without pancreatic cancer (autopsy)** | | | |
| --- | --- | --- | --- | --- | --- | --- | --- |
| **ID** | **Age y** | **Gender** | **Mercury** | **ID** | **Age y** | **Gender** | **Mercury** |
| P01 | 39 | Female | + a,d | A01 | 35 | Male | 0 |
| P02 | 39 | Male | 0 | A02 | 35 | Female | 0 |
| P03 | 45 | Male | + a | A03 | 37 | Female | 0 |
| P04 | 46 | Male | ++ a,d,D | A04 | 38 | Male | 0 |
| P05 | 48 | Female | + | A05 | 38 | Female | + |
| P06 | 50 | Female | + | A06 | 38 | Female | 0 |
| P07 | 58 | Male | 0 | A07 | 39 | Male | ++ |
| P08 | 58 | Male | ++ | A08 | 39 | Male | + |
| P09 | 59 | Male | 0 | A09 | 40 | Female | 0 |
| P10 | 59 | Male | + a,d | A10 | 40 | Female | + |
| P11 | 59 | Male | 0 | A11 | 41 | Male | 0 |
| P12 | 60 | Male | 0 | A12 | 41 | Male | 0 |
| P13 | 65 | Male | + | A13 | 44 | Female | 0 |
| P14 | 66 | Female | 0 | A14 | 45 | Male | 0 |
| P15 | 66 | Male | + | A15 | 49 | Male | 0 |
| P16 | 67 | Male | 0 | A16 | 61 | Male | 0 |
| P17 | 68 | Female | + a | A17 | 70 | Male | 0 |
| P18 | 68 | Female | 0 | A18 | 72 | Female | 0 |
| P19 | 69 | Female | + | A19 | 75 | Male | 0 |
| P20 | 69 | Male | + | A20 | 78 | Female | 0 |
| P21 | 70 | Male | 0 | A21 | 80 | Female | + |
| P22 | 71 | Male | ++ a | A22 | 80 | Male | 0 |
| P23 | 71 | Male | + | A23 | 81 | Female | 0 |
| P24 | 72 | Female | + | A24 | 85 | Male | 0 |
| P25 | 72 | Male | + a | A25 | 87 | Female | 0 |
| P26 | 72 | Male | 0 | A26 | 95 | Female | 0 |
| P27 | 74 | Female | + | A27 | 95 | Female | + |
| P28 | 76 | Female | + a | A28 | 95 | Male | 0 |
| P29 | 77 | Female | 0 | A29 | 95 | Male | 0 |
| P30 | 77 | Female | 0 | A30 | 95 | Male | 0 |
| P31 | 77 | Male | + a | A31 | 96 | Female | 0 |
| P32 | 78 | Female | 0 | A32 | 96 | Male | 0 |
| P33 | 78 | Male | 0 | A33 | 96 | Female | 0 |
| P34 | 79 | Male | 0 | A34 | 96 | Female | 0 |
| P35 | 80 | Male | 0 | A35 | 97 | Female | 0 |
| P36 | 80 | Male | + | A36 | 97 | Female | 0 |
| P37 | 81 | Male | 0 | A37 | 104 | Female | 0 |
| P38 | 81 | Male | 0 | A38 | 104 | Female | 0 |
| P39 | 82 | Male | 0 |  |  |  |  |
| P40 | 83 | Male | 0 |  |  |  |  |
| P41 | 83 | Male | + |  |  |  |  |
| P42 | 83 | Female | + |  |  |  |  |
| P43 | 83 | Female | ++ a,d |  |  |  |  |
| P44 | 86 | Male | 0 |  |  |  |  |
| P45 | 87 | Male | + a |  |  |  |  |

A: autopsy sample identity (ID) number, P: pancreatectomy sample identity (ID) number, y: years. 0: no mercury-containing islets, +: <50% of mercury-containing islets, ++: ≥50% of mercury-containing islets, a: acinar cell mercury , d: small duct mercury, D: large duct mercury
